# A peculiar case of *Campylobacter jejuni* attenuated aspartate chemosensory mutant, able to cause pathology and inflammation in avian and murine model animals

**DOI:** 10.1038/s41598-018-30604-5

**Published:** 2018-08-22

**Authors:** L. E. Hartley-Tassell, C. J. Day, E. A. Semchenko, G. Tram, L. I. Calderon-Gomez, Z. Klipic, A. M. Barry, A. K. Lam, M. A. McGuckin, V. Korolik

**Affiliations:** 1 Institute for Glycomics, Griffith University, Gold Coast, Queensland 4222 Australia; 2 School of Medicine and Menzies Health Institute Queensland, Griffith University, Gold Coast, Queensland 4222 Australia; 3 Translational Research Institute, Mater Research, Brisbane, Queensland 4102 Australia

## Abstract

An attenuated *Campylobacter jejuni* aspartate chemoreceptor *ccaA* mutant caused gross pathological changes despite reduced colonisation ability in animal models. In chickens, the pathological changes included connective tissue and thickening of the mesenteric fat, as well as the disintegration of the villus tips in the large intestine, whereas in mice, hepatomegaly occurred between 48–72 hours post infection and persisted for the six days of the time course. In addition, there was a significant change in the levels of IL-12p70 in mice infected with the *C*. *jejuni ccaA* mutant. *CcaA* isogenic mutant was hyper-invasive in cell culture and microscopic examination revealed that it had a “run” bias in its “run-and-tumble” chemotactic behaviour. The mutant cells also exhibited lower level of binding to fucosylated and higher binding to sialylated glycan structures in glycan array analysis. This study highlights the importance of investigating phenotypic changes in *C*. *jejuni*, as we have shown that specific mutants can cause pathological changes in the host, despite reduction in colonisation potential.

## Introduction

*Campylobacter* infections are one of the top four key global causes of bacterial gastroenteritis world-wide^1^. *Campylobacter jejuni* is widely regarded as a commensal of the avian gut, and chickens, specifically, are considered to be the major vector for this zoonotic illness^1–3^. In humans, however, infection results in severe gastroenteritis in which the pathology presents as severe active inflammation of the intestinal mucosa with an influx of phagocytes^4–6^.

There are several colonisation factors, which contribute to the infection of the human gastrointestinal tract and colonisation of the avian gut as a commensal. These factors include chemotaxis, motility, capsule formation, two-component regulatory systems, invasion and iron regulation (reviewed in^7^). Specifically, chemotaxis and motility have been implicated in colonisation and virulence of *C*. *jejuni*^8,9^. During infection in humans, *C*. *jejuni* invades and traverses the intestinal epithelium^5,10^, causing disruption to the epithelium and gaining access to the basal side^11^. Infection also stimulates the innate immune system with upregulation of the inflammatory cytokines Il-1β, IL-8 and nitric oxide^12^.

The study of host-pathogen interactions of *C*. *jejuni* suffer due to a paucity of suitable animal models which accurately mimic human campylobacteriosis. However, animal models of colonisation and infection, such as chickens^13–15^ and mice^16–18^, are commonly used to elucidate interactions of *C*. *jejuni* with its hosts. The use of mice however, as a campylobacter infection model has proven problematic, as most wild-type laboratory mouse strains are susceptible only to a short transient infection of the gut with no discernible symptoms. In order to elicit a disease phenotype upon infection, mouse models have subsequently been adapted by employing SIGIRR-deficient (−/−) or IL-10(−/−) mice^19–21^. However, these mice are immunocompromised and the establishment of infection and disease is unrealistic compared to the immunocompetent response. Despite these limitations, murine models are extensively used to study all aspects of *C*. *jejuni* colonisation^22,23^.

Alternatively, study by McAuley *et al*.^18^, showed that 129/SvJ background mice were susceptible to persistent colonisation by *C*. *jejuni* which localised in digestive and systemic organs of these mice^18^. While 129/SvJ mice are useful as a colonisation and not a disease model, similar to that with other murine models, they are immunocompetent and can provide useful information of a mammalian immune response to colonisation with *C*. *jejuni* and its isogenic mutants.

One major contrast between the human and avian hosts, is that *C*. *jejuni* infection in chickens does not typically lead to the same symptoms and pathological inflammatory response as in humans^24^. The physiological reasons for this are yet to be elucidated. There is however conflicting evidence as to whether campylobacters can adhere to or invade the chicken gut, and if indeed campylobacters are a commensal^2,25^. *C*. *jejuni* is commonly found in the mucus layer, and especially in the deep crypts of the caecum^8^, and some evidence suggests that campylobacters have the ability to traverse the intestinal epithelium, as bacteria have been recovered in the liver and lungs of young chicks^26^. Recent studies have also suggested that *C*. *jejuni* infection in the chicken gut initiates an innate immune response^27^ which has also been shown in avian cell lines with stimulation of pro-inflammatory cytokine response^28^.

The differences in *C*. *jejuni* relationship with its human and animal hosts had been a subject of intense speculation with little evidence to support any of the theories. However, genes involved in flagella, motility and chemotaxis, as either receptors or other elements of chemotaxis machinery, have been shown to be important for colonisation of the gastrointestinal tract of chickens^6,15^. Additionally, chemotaxis genes were shown to be differentially expressed in *C*. *jejuni* cells isolated from a chicken host, as well as the genes involved in electron transport and the central metabolic pathways^29^. Changes in sensory receptor gene expression have also been described for *C*. *jejuni* strains 11168-O, 11168-GS, 81116 and 520 when isolated from different sources, including the intestine of mice and chickens^30^.

We have previously shown that a mutation of the sensory domain of the aspartate chemosensor CcaA of the original (Skirrow) isolate of *C*. *jejuni*, NCTC 11168-O, resulted in a “run” chemotactic motility bias, a reduced ability to colonise the gastrointestinal tract of chickens and increased efficiency in invasion of Caco-2 cells when compared to the 11168-O wild type and *ccaA*^−/+^ complemented strains^31^. Since CcaA appears to play an important role in colonisation of the chicken, the effect of a mutation in *ccaA* of *C*. *jejuni* 11168-O on interaction with both the avian and mammalian hosts needed to be assessed further.

In this study, we describe systematic analysis of the effect of *C*. *jejuni* 11168-O CcaA mutation on the interaction of the bacteria with avian and mammalian hosts during different stages of colonisation as compared with the parental wild type strain. Here we report the first observation and analysis of abnormal gross pathology of the liver in a murine model, and thickening of the mesenteric fat around the intestinal connective tissue in the chicken model, following infection with *C*. *jejuni ccaA* mutant, but not when infected with wild type or complemented mutant strain, as we described previously^31^. We further report the of the isogenic mutant’s ability to bind simple and complex glycans as well as the expression profiles of the pro-inflammatory cytokines in the avian and murine model were investigated in response to infection with *C*. *jejuni* 11168-O and its isogenic *ccaA* mutant

## Results

### Colonisation potential in the murine model

**Figure 1:**
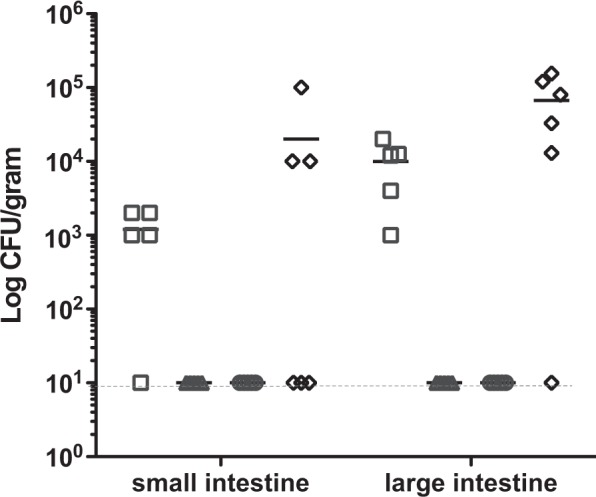
Bacterial load for the small intestine and large intestine at day 6 post-inoculation. Data is displayed as log cfu per gram of intestinal content, n = 5. 11168-O (□), 11168-O*ΔccaA::cat* (Δ), negative non-infected control (○), competition 11168-O WT and *ΔccaA::cat* (◊). Bar indicates average cfu/gram for each group. <10 cfu/gram of intestinal content was detected for 11168-O*ΔccaA::cat* and mock infected. Broken line indicates level of sensitivity.

To analyse the colonisation deficiency of the *C*. *jejuni* aspartate receptor mutant, the colonisation potential of *C*. *jejuni* 11168-O and 11168-O*ΔccaA::cat* was compared in 129/SvJ mice over a 6 day period. The post-mortem analyses of mice intestinal content on day 6 post-inoculation showed that the average bacterial load in the large intestine of mice infected with wild type 11168-O was 1.2 × 10^4^ cfu/g, while mice infected with 11168-O*ΔccaA::cat* had an average bacterial load of 1 × 10^1^ cfu/g. This indicated a 3-log reduction in presence of 11168-O*ΔccaA::cat* cells in the large intestine, when compared to that of 11168-O wild-type (p < 0.001) (Fig. 1). It should be noted that infection with 11168-O*ΔccaA::cat* resulted in a reduction in colonisation for the avian host of 1.5-log, (reported previously^31^). Selective plating of the viable bacteria recovered from the post-mortem intestinal content from the co-infection of the mice with wild-type and mutant CcaA, showed that >98.8% of bacteria present in the small and large intestines was wild-type 11168-O (p > 0.25) on day 6p.i. The average bacterial load in both the small and large intestine for the 11168-O wild-type as sole inoculum and for co-infection with the *ccaA* mutant were not statistically different at day 6p.i. (students T-test, p > 0.05).

### Post-mortem analysis of affected murine tissues

Importantly, when mice were sacrificed on day 6p.i., prominent gross pathological differences were observed in the mice infected with 11168-O*ΔccaA::cat*, when compared to those infected with 11168-O wild-type. Despite the reduced bacterial load, the liver of the mice infected with 11168-O*ΔccaA::cat* appeared noticeably larger. The livers of mice infected with 11168-O*ΔccaA::cat* were significantly larger in weight (p < 0.01) when compared to the other groups with mean weight of 1.3 g on day 6p.i., a 60–80% increase in liver weight. Upon dissection of the gastrointestinal tract, the mesenteric lymph nodes were visually more prominent in mice infected with 11168-O*ΔccaA::cat* when compared to 11168-O wild-type. Additionally, the Peyer’s patches were enlarged along the entire length of the intestine, for both 11168-O and 11168-O*ΔccaA::cat* (not shown).

### Four-day time course murine infection trial

To ascertain the progression of the pathological changes in the mice, induced by 11168-O*ΔccaA::cat* isogenic mutant, a time course experiment was conducted at time points of 24, 48, 72 and 96 hours post inoculation. (Table S1). The bacterial counts from lungs, liver and spleen for mice singly infected with 11168-O or 11168-O*ΔccaA::cat* were not statistically different (p < 0.05), and there was no difference in bacterial load in the duodenum, small or large intestine (p > 0.05).

### Post-mortem analysis of affected murine tissues, 4 day time course trial

**Figure 2:**
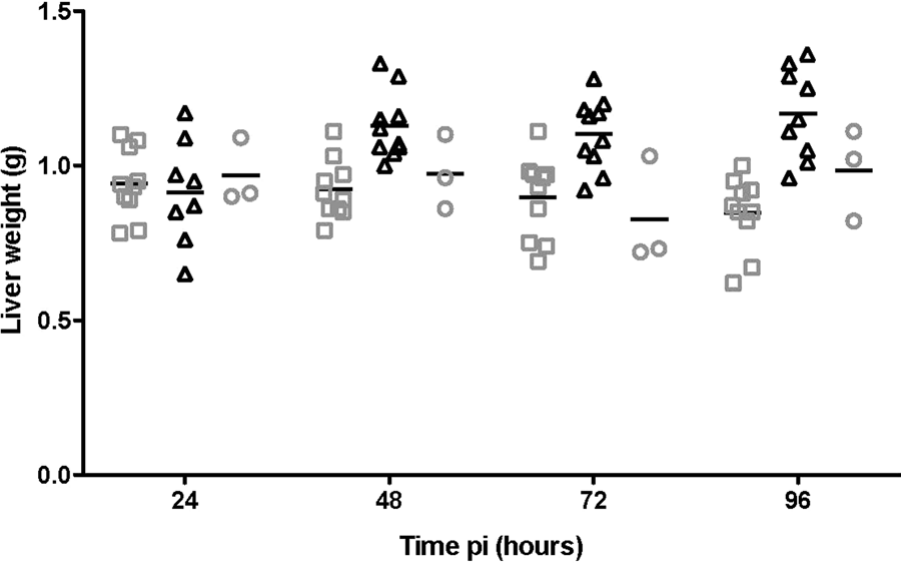
Liver weights in mice. The mean of each group is shown as a bar. 11168-O (□), 11168-O*ΔccaA::cat* (Δ), negative non-infected control (○). Average weight is shown as a bar for each group at each time point. *p < 0.05 ANOVA, 11168-O*ΔccaA::cat* weights are greater than 11168-O and negative groups at 48, 72 and 96 hours post inoculation. N = 3–10 mice.

Further post-mortem analysis of mice during the time course trial, showed no difference in liver weight (as a percentage of body weight) at 24 h p.i. when comparing the mock-infected control, 11168-O and 11168-O*ΔccaA::cat* inoculated animals (p > 0.18). However, at 48 h post-inoculation, the liver weight for 11168-O*ΔccaA::cat* inoculated group was significantly higher than those inoculated with 11168-O and negative controls (p < 0.02, Fig. 2). For the mice inoculated with 11168-O*ΔccaA::cat*, an average increase of 23% in relative liver size was observed after 48 h (Fig. 2). In order to determine if this phenomenon persisted long-term mice, mice were sacrificed on day 14p.i. There was no statistical difference in the relative liver size for all groups, indicating that this pathology was only present during the acute infection period (data not shown).

### Histopathological analysis of the systemic and digestive organs of mice infected with *C*. *jejuni* 11168-O or 11168-O*ΔccaA::cat*

To further examine the pathological differences seen in the mice infected with isogenic mutant strain of *C*. *jejuni* 11168-O, histological samples for the systemic and digestive organs were taken for each mouse at every time point (24–96 h p.i.), and scored blindly according to tissue pathology using haematoxylin and eosin (H&E) staining.

Pathological differences were noted in the severity of inflammatory cells infiltration into the crypts of the mid-colon, which was found to be significantly higher in 11168-O*ΔccaA::cat* infected mice when compared to that of the 11168-O group and non-infected control groups (p < 0.05) as shown in Table S2. Goblet cell loss within the small intestine was observed in all groups, in conjunction with some goblet cell hyperplasia. There was, however, no statistical difference in the extent of goblet cell loss or hyperplasia dependent on inoculum or time point (p > 0.05). Paneth cells were prominent in 11168-O*ΔccaA::cat-*infected mice at 24 h (1/10) and 48 h (4/10), this, however, was not statistically significant. There was also evidence of *C*. *jejuni* present within the goblet cells of the mid and distal colon in 11168-O*ΔccaA::cat-*infected mice, however this was not consistently observed within the group (7/40). The liver was scored in terms of nuclei enlargement, blood vessel dilation and granulation. There were no significant differences between the groups. No significant differences were noted for the lungs, liver and spleen or in the tissues of proximal or distal colon obtained from mice infected with 11168-O*ΔccaA::cat*, 11168-O or non-infected controls.

### Single-cell tracking microscopic analysis

Unlike other attenuated chemoreceptor mutants previously reported^32,33^, the mutation of the aspartate receptor CcaA resulted in an increase, rather than the decrease, in both adherence and invasion of cultured Caco-2 cells. This unusual phenotype of the CcaA mutant could be related to a “run” chemotactic behaviour was investigated further. Single-cell tracking of wild-type, mutant and complemented *C*. *jejuni* isogenic strains showed that the mutant had increased linear displacement over 1 second in time (Table S3, p < 0.005) confirming a run-biased phenotype, compared to 11168-O and the *ccaA*^−/+^ complement which both showed tumbling and running phenotypes. This was further confirmed by a swarm plate assay (Table S3).

### Colonisation potential in the avian model

A competitive co-infection assay was used to determine the fitness of the isogenic mutant 11168-O*ΔccaA::cat*. Co-infection of chickens with both *C*. *jejuni* 11168-O and 11168-O*ΔccaA::cat*, showed that only the wild type strain could be could be recovered after 24, 48, 72 and 96 h following inoculation (p < 0.01).

**Figure 3:**
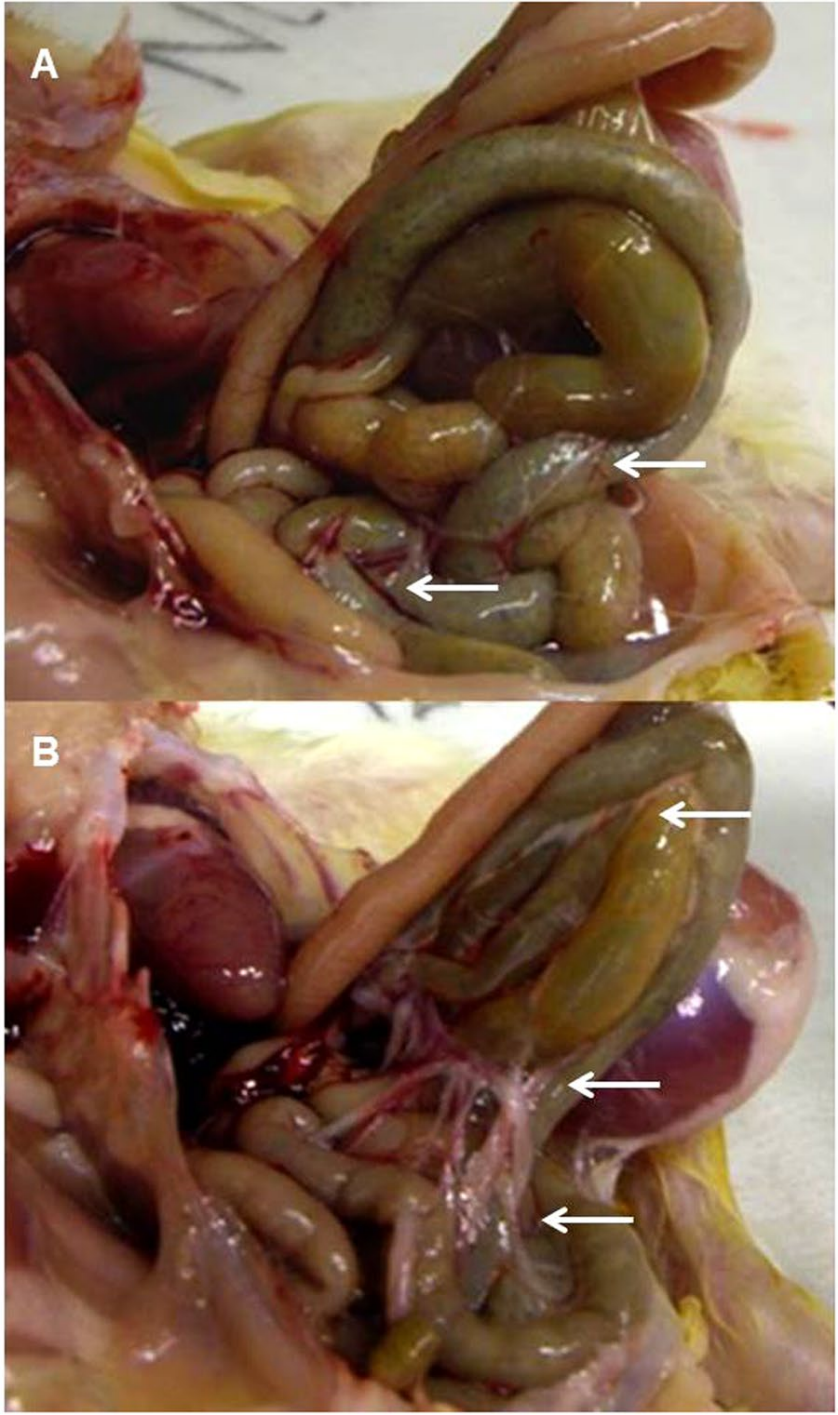
Pathology of the chick gut day 5 post inoculation. (**A**) Chick infected with *C. jejuni* 11168-0. (**B**) Chick infected with *C. jejuni* 11168-0*ΔccaA::cat*. Arrows show areas for visual comparison of the mesenteric fat surrounding the intestines.

It was also noted that when the chickens were sacrificed on day 5 post-inoculation, similar to that seen in the murine host, prominent gross pathological differences were observed in the chickens infected with 11168-O*ΔccaA::cat*, when compared to those infected with 11168-O wild-type or the *ccaA*^−/+^ complement. Despite the reduced bacterial load, the intestines were surrounded by fluid and rope-like thickening of the mesenteric fat (Fig. 3).

### Four-day time course avian colonisation trial

To ascertain the time of appearance of the pathological changes induced by 11168-O*ΔccaA::cat* isogenic mutant, a time course experiment was then conducted whereby groups of ten day-old chicks, inoculated with *C*. *jejuni* mutant and wild type strains, were sacrificed and dissected every 24 h, over a 96 h period. Histological examination revealed thickening of the mesenteric fat at 72 h with increasing evidence at 96 h. Interestingly, no diarrhoea was noted for this group of chickens. In contrast, for the chicks infected with 11168-O*ΔccaA::cat*, stools appeared firm and pellet-like in contrast to typical loose cloacal secretions normally observed for chickens colonised with wild type *C*. *jejuni* strains, including 11168-O.

The severity and extent of infection of *C*. *jejuni* in systemic organs was assessed by enumeration of bacterial load within each chicken at time points of 24, 48, 72 and 96 h p.i. (Table S4). Despite the changes in gross pathology, there was no statistical difference between the overall bacterial counts in the systemic organs, small or large intestine for animals infected with 11168-O and 11168-O*ΔccaA::cat* isogenic strains at any time point. The average bacterial counts in the liver for chickens infected with 11168-O*ΔccaA::cat* was 2.5 × 10^2^ cfu/gram, when compared to 11168-O which was 1 × 10^2^ cfu/gram. There was no statistical difference in the bacteria present in the for chickens infected with 11168-O or 11168-O*ΔccaA::cat*.

### Post-mortem analysis of affected avian tissues

**Figure 4:**
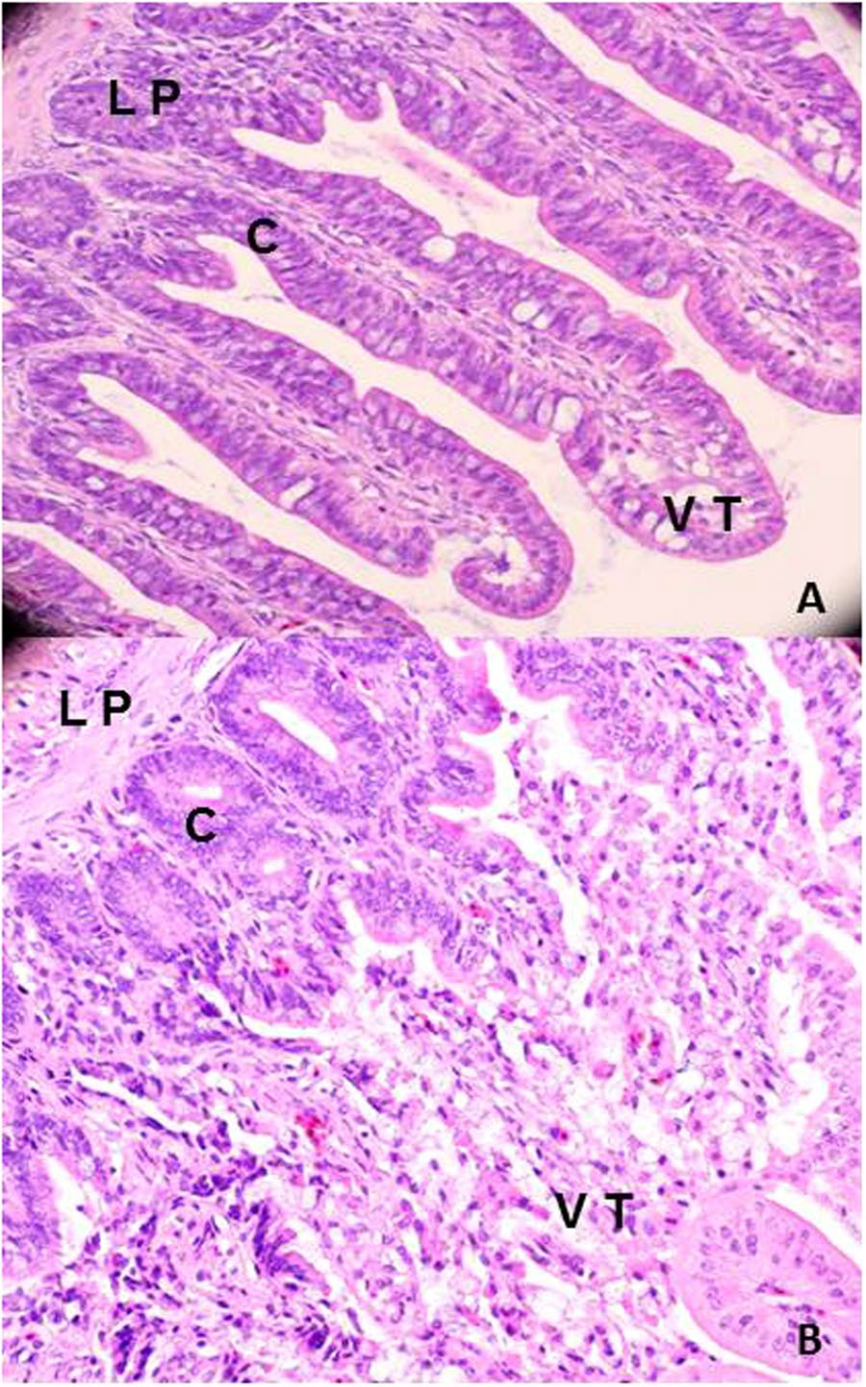
Haematoxylin and eosin (H&E) stained cross sections of the large intestine of chickens infected with 11168-O*ΔccaA::cat* or 11168-O. (**A**) H&E stained section of large intestine of chicken infected with 11168-O. (**B**) H&E stained section of large intestine of chicken infected with 11168-O*ΔccaA::cat*. LP: Lamina propria, C: Crypt of villi, VT: Villi tip.

Since gross pathological differences were seen in chickens, the remainder of each tissue collected from the animals during the time course experiment was examined histologically. The H & E stained sections of the tissues were scored blindly according to tissue pathology, Table S5. There was no significant difference in pathology between 11168-O*ΔccaA::cat*, 11168-O and non-infected controls for the lungs, liver, spleen, and small intestines. However, there was a statistical difference in the extent of villus epithelium shedding observed in the large intestine of chickens infected with 11168-O*ΔccaA::cat* which was significantly higher than that for 11168-O or non-infected controls (p < 0.02). Epithelial shedding was characterised by disintegration of the tips of the villi as shown in Fig. 4.

### Analysis of adhesion and invasion ability of 11168-O and 11168-O*ΔccaA::cat* mutant in cell culture assays

**Figure 5:**
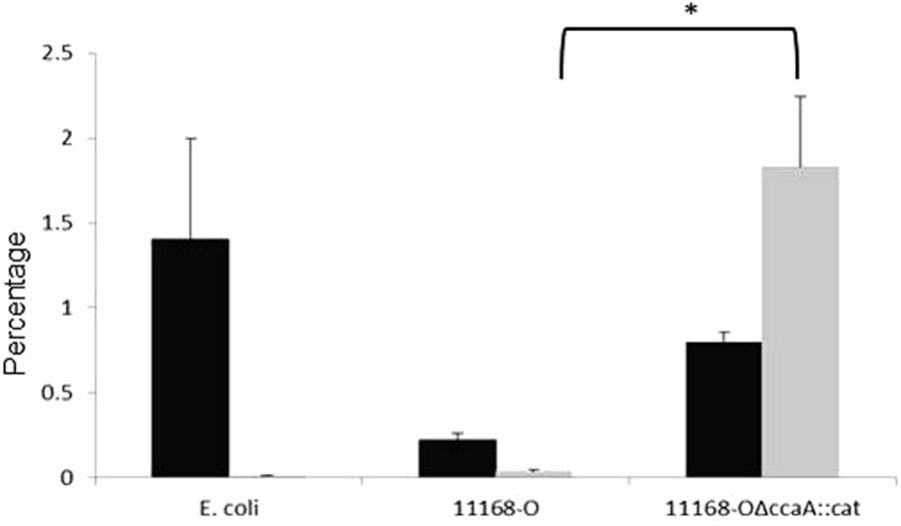
Adherence and Invasion of *C. jejuni* of *in vitro* cell culture model using A549 lung epithelial cells. Adherence analysis (black) and invasion (grey) of *E. coli* DH5α, *C. jejuni* 11168-0 and 11168-O*ΔccaA::cat*. Results are presented as mean ± SEM adherence or invasion from six to nine wells of a 24-well plate. Two tailed t-test showed no significant increase in adherence of 11168-O*ΔccaA::cat* compared to wild-type, p = 0.058, and a significant increase in invasion of 11168-O*ΔccaA::cat* compared to wild-type (Student’s T-test, p < 0.001).

Since high numbers of *C*. *jejuni* were recovered from the chicken lung homogenate, the ability of *C*. *jejuni* to adhere and invade a lung cell line was investigated. A549 human lung adenocarcinoma cell line was used, noting that the avian lung differs to the mammalian lung, as it does not have alveoli and some surface glycans on the cells also differ. The results from the *in vitro* cell culture using A549 cells showed that 11168-O was less adherent (0.22%) when compared to 11168-O*ΔccaA::cat* (1.15%) although the difference was not significant (p = 0.058). There was, however, a statistically significant difference between the invasion ability of 11168-O and 11168-O*ΔccaA::cat*, showing that 11168-O*ΔccaA::cat* was highly invasive, with 2.24% of adhered bacteria invading the cells (p < 0.001), compared to 0.003% for 11168-O wild-type strain, as shown in Fig. 5.

**Figure 6:**
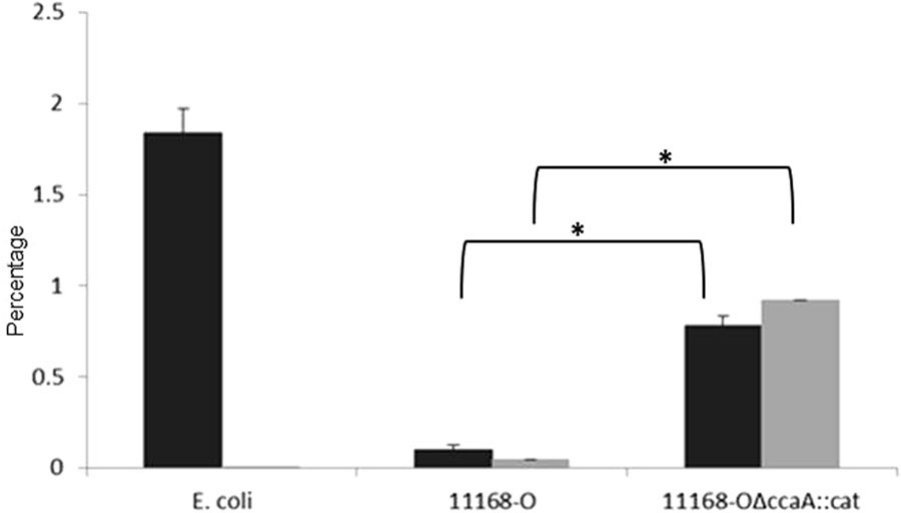
Adherence and Invasion of *C. jejuni* of *in vitro* cell culture model using Hep-G2 liver cells. Black) Adherence analysis (black) and invasion (grey) of *E. coli* DH5α, *C. jejuni* 11168-0 and 11168-O*ΔccaA::cat*. Results are presented as mean adherence from six to nine wells of a 24-well plate. Standard errors are shown as bars above the mean. Two tailed t-test showed a significant increase in adherence of 11168-O*ΔccaA::cat* compared to wild-type, (p < 0.01) and a significant increase for invasion for the *ccaA* mutant compared to wild-type (p < 0.001).

Since viable *C*. *jejuni* 11168-O*ΔccaA::cat* was recovered from mice livers with hepatomegaly, the ability of *C*. *jejuni* to adhere to and invade a liver cell line was investigated. The adherence levels of 11168 and 11168-O*ΔccaA::cat in vitro* cell culture of Hep-G2, human hepatocellular liver carcinoma cell line were higher for 11168-O*ΔccaA::cat* at 0.78% when compared to that for 11168-O at 0.1% (p < 0.01, Fig. 6). The isogenic mutant 11168-O*ΔccaA::cat* was also more invasive, with 0.925% of adhered bacteria invading the cells (p < 0.001), compared to 0.0457% for 11168-O.

### Glycan binding profile of *C*. *jejuni* isolated from avian hosts

Glycan array analysis of the bacterium isolated from the caecum of chicks was performed in order to determine if the host glycan binding profile has altered with the mutation of the CcaA. Glycan array analysis of whole cell *C*. *jejuni* was performed as previously described by Day *et al*., 2013, whereby glycans (Table S6) were tested for binding by *C*. *jejuni* 11168-GS and 11168-O isolated from the caecal content of chicks at day 5p.i. by IMS^34^. The binding specificities of 11168-O and 11168-O*ΔccaA::cat* were compared and only statistically significant differences (p < 0.05) are described in Table 1. *C*. *jejuni* 11168-O*ΔccaA::cat* had reduced ability to bind type II fucosylated structures, including lacto-*N*-fucopentaose II, Lewis^y^, blood group H type II trisaccharide and monofucosyllacto-*N*-hexaose III. The mutant did however display significantly stronger binding to both 3′-sialyllactosamine and 6′-sialyllactosamine compared to the wild type 11168-O.

**Table 1:** Glycan binding profile of *C. jejuni* isolated from avian hosts.

Compound	Strain	Structure
β1-6 Galactosyl-*N*-acetyl glucosamine	WT	ASialo GM1
Lacto-*N*-neohexaose	WT	Type I
Lacto-*N*-hexaose	WT	Type II
GalNAcα1-3Galβ1-4Glc	CcaA	Type I
β1-2 *N*-Acetylglucosamine-mannose	CcaA	N-type glycan
α1-3,α1-6-Mannobiose	WT	BiMan
α1-3,α1-3α,α1-6-Mannopentaose	CcaA	OligoMan
Lacto-*N*-fucopentaose II	WT	Type II-fucosylated
Lewis^y^	WT	Type II-fucosylated
Blood group H type II trisaccharide	WT	Type II-fucosylated
Monofucosyllacto-*N*-hexaose	WT	Type II-fucosylated
3′-Sialyllactosamine	CcaA	Type II-sialylated
6′-Sialyllactosamine	CcaA	Type II-sialylated
Colominic acid	WT	Oligo Sialic acid
ΔUA-GlucNS-6S	CcaA	Digests of GAGs
ΔUA-GalNAc-4S,6S (Delta Di-disE)	CcaA	Digests of GAGs

WT indicates significantly stronger binding of *C. jejuni* 11168-O to the specific glycan compared to 11168-O*ΔccaA::cat*. CcaA indicates significantly stronger binding of 11168-O*ΔccaA::cat* to the specific glycan compared to 11168-O.

### Immune response in the murine model - Mouse CBA inflammatory cytokine array

**Figure 7:**
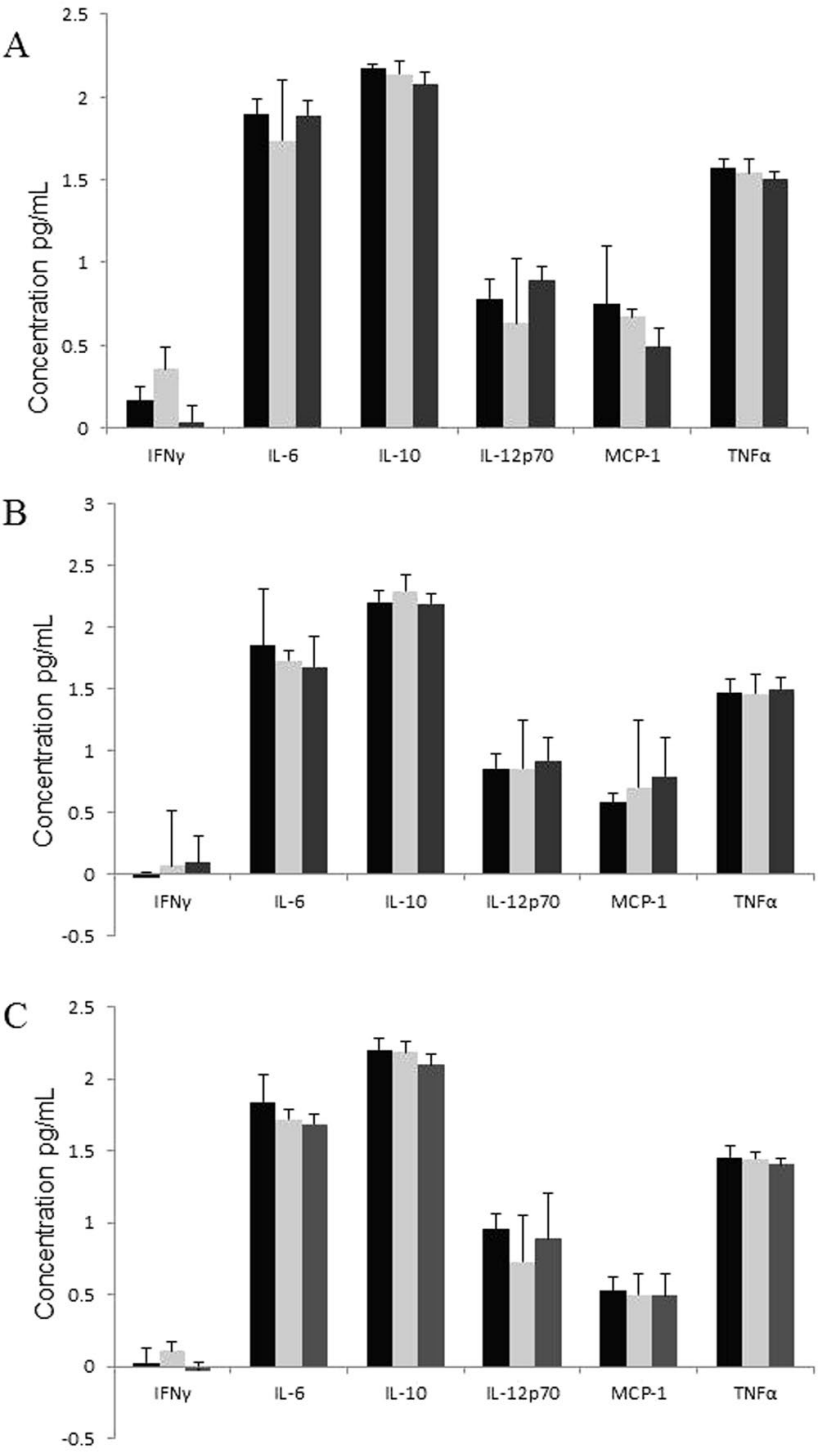
Proinflammatory cytokine concentrations in the small intestine of mice infected with *C. jejuni*. Proinflammatory cytokine concentrations were determined by CBA array at time points of (**A**) 24 h, (**B**) 48 h and (**C**) 72 h, n = 7. Cytokine levels of non-infected (black), 11168-O wild-type (light grey) and 11168-O*ΔccaA::cat* (mid grey). No statistical differences were noted (ANOVA, p < 0.05).

In order to analyse the differences in the immune response triggered by the wild type and the aspartate mutant of *C*. *jejuni*, inflammatory cytokine levels were examined. A significant change was seen in the levels of IL-12p70 within the *C*. *jejuni* 11168-O*ΔccaA::cat* group, with higher levels detected at 48 h and 72 h when compared to the levels at 24 h p.i.(p < 0.05). There appeared to be no other differences in inflammatory cytokine levels between the non-infected control animals and those infected with either the *C*. *jejuni* 11168-O or *C*. *jejuni* 11168-O*ΔccaA::cat* at 24 h, 48 h or 72 h p.i. (Fig. 7A–C).

### Quantitation of inflammatory cytokine expression in chicken by qPCR

**Figure 8:**
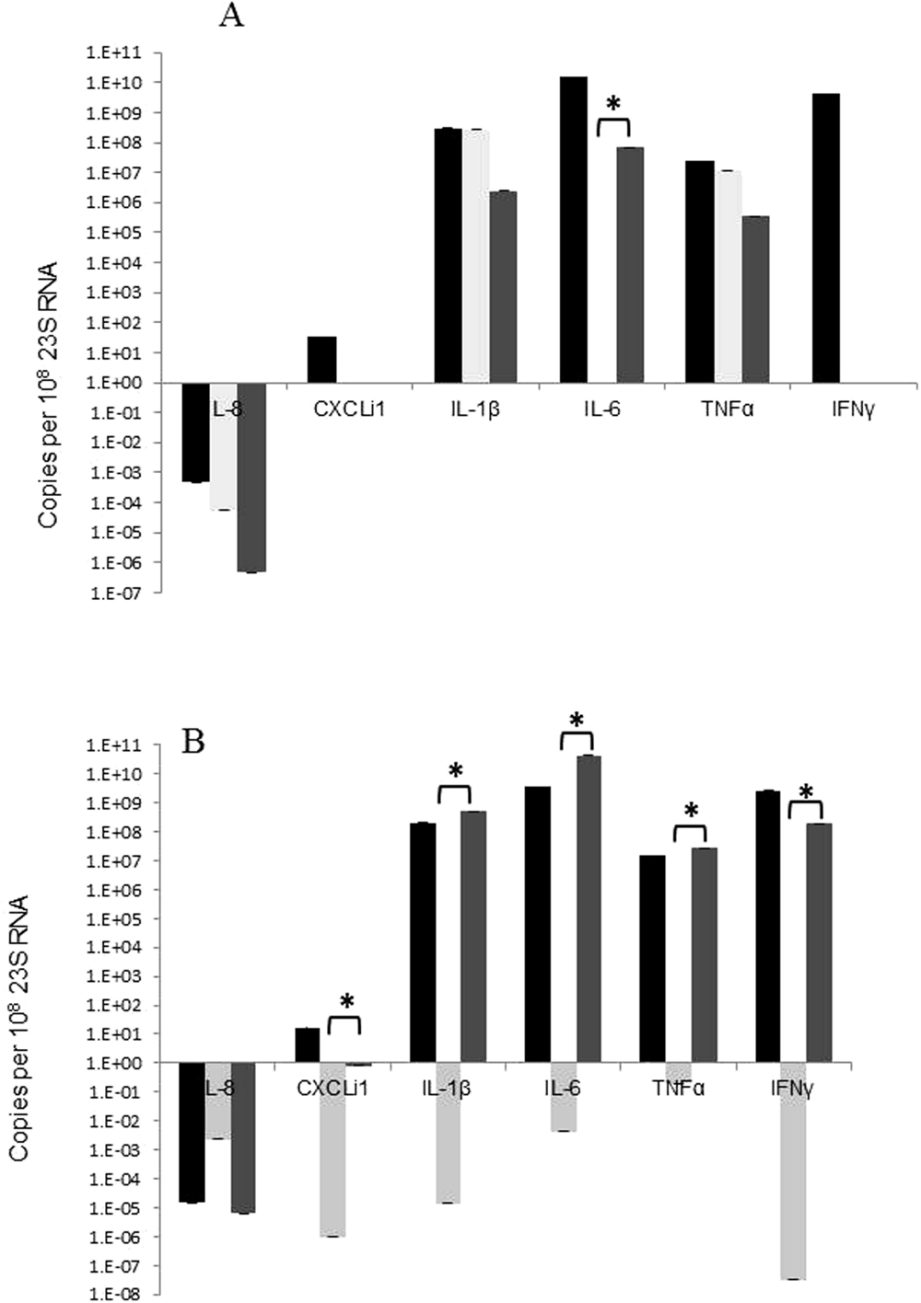
Cytokine levels in chickens determined by qPCR. Relative expression levels of the inflammatory cytokines in chicken large intestines at 24 h (**A**) or 96 h (**B**), non-infected control (Black), 11168-O WT (light grey) and 11168- O*ΔccaA::cat* (mid grey), n = 5. Expression is standardised and the scale is shown in log (copies per 10^8^ 28S). Statistically significant differences are indicated by *, Student’s T-test (p < 0.01).

Similar to the murine host, the inflammatory response of avian host was also examined. RNA extracted from the large intestine of the chickens at 24 h and 96 h time points, cDNA was isolated and qPCR was performed investigating the levels of the inflammatory cytokines IL-8, IL-1β, IL-6, CXCLi1, TNFα and IFNγ. At 24 h p.i., the cytokine levels of IL-8, IL-1β, CXCLi1 and TNFα in chicks infected with both *C*. *jejuni* 11168-O and the *ccaA* mutant showed no significant change in expression. There was however a significant decrease in expression of IFNγ for both WT and mutant infected chicks (Fig. 8A).

At 96 h p.i., the expression of IL-1β, IL-6, TNFα and IFNγ in WT infected chicks was significantly less than the levels determined in the uninfected control group. The expression of these cytokines IL-1β, IL-6, TNFα and IFNγ were all significantly higher for the mutant *C*. *jejuni* 11168-O*ΔccaA::cat* group than for the WT control group, and for IL-1β and IFNγ the expression is also statistically greater than the uninfected control levels (Fig. 8B).

At no time point was there any significant change in the expression of IL-8 cytokine or the CXCLi1 chemokine.

### Expression of porA, peb1A cdtA, cdtB and cdtC

To ascertain if the “run” phenotype of the aspartate receptor mutant was responsible for the observed pathological changes in the infected animals, the expression of known virulence genes was investigated. The analysis of gene expression profiles of *porA*, *peb1A* and *cdtABC* in *C*. *jejuni* cells isolated directly from the chicken caeca and from mouse intestinal tract (by IMS) showed that there was no difference in expression in *porA* or *peb1A* for 11168-O when compared to 11168-O*ΔccaA::cat* when grown at 37 °C or 42 °C *in vitro* (core temperature of mammals and avians respectively). Interestingly, when grown in the chicken host, the expression of *porA* was not detectable in the wild type strain 11168-O whereas *porA* was highly expressed in the isogenic mutant 11168-O*ΔccaA::cat*. In the mouse host, however, both were equally expressed. In 11168-O*ΔccaA::cat*, there was no statistical difference in the level of expression of *porA and peb1A in vivo* when compared to *in vitro* at 42 °C (Fig. S1).

Expression of *cdtA and C*, but not *B* was significantly higher in 11168-O*ΔccaA::cat* when compared to 11168-O when it was culture *in vitro* at 37 °C (p < 0.05; Fig. S1). The expression of *cdtA* in both 11168-O and 11168-O*ΔccaA::cat* isolated from chickens was up-regulated. In contrast *CdtA* and *B* expression in 11168-O was up-regulated *in vivo* compared to *in vitro* culture grown at 42 °C, while *cdtB* and *C* were up-regulated *in vitro* for 11168-O*ΔccaA::cat*, when gene expression levels were compared to those observed for 11168-O at 42 °C (p < 0.05). Most importantly, however, the expression of *cdtABC in vivo*, in a chicken and mouse host did not show statistical difference in expression (p > 0.05). This indicates that although the overall expressions of the three CDT subunits results showed that gene expression is variable at different temperatures, and expression is regulated *in vivo*. These genes are unlikely to be involved in the observed gross pathology.

## Discussion

Mutational analyses are a classical method for determining gene function and attenuation of virulence, particularly when assessing potential antimicrobial targets. *C*. *jejuni*, a commensal in poultry and pathogen in humans, has also been a subject to many such analyses.

Previous avian colonisation studies using various *C*. *jejuni* chemosensory pathway mutants, did not report any pathological changes^13,32,33^. This study revealed that despite attenuation of colonisation in both the murine and avian models, gross pathological changes were observed in both mice and chickens infected with the *C*. *jejuni* aspartate chemoreceptor mutant *ccaA*, including connective tissue and fat roping in chickens and hepatomegaly in mice. The ability of the *ccaA* mutant to produce pathological changes in animal hosts is a novel and unusual observation.

Specifically, the appearance of intestinal roping was noted at 72 h p.i. along with the disintegration of the tips of the villi in the large intestine of chickens infected with the *ccaA* mutant, which persisted throughout the entire 96 hour examination period and correlated to the chicken stools appearing unusually firm and pellet-like. This too was unexpected as it has been well established that in humans *C*. *jejuni* infection is characterised by diarrhoea whereas in the chickens, *C*. *jejuni* colonisation is asymptomatic.

The hardened appearance of the faeces in chickens infected with the *ccaA* mutant may be due to dis-regulation of CDT toxin production, causing the faeces to become drier and more solid, compared to normal faeces. In humans, the cytolethal distending toxin, CDT, is thought to destroy the mucosal epithelium, cause secretory diarrhoea and necrosis of the colonic epithelium^35^. Although in chickens, *cdt* toxin genes are expressed, no symptoms of diarrhoea are usually observed^36^. The expression of the CDT toxin genes, however, may contribute toward the usually observed appearance of semi-solid chicken faeces. This change in the consistency of the cloacal secretions may contribute to the damage to the villus epithelium in the large intestine as was seen in the histological sections. The *cdtA* gene expression by *ccaA* isogenic mutant cells isolated from chickens was up-regulated as was the expression of *cdtB* in the mutant cells isolated from the mouse. This was in agreement with a previous study showing that in chickens the *cdtA/C* subunits could be up-regulated^34^. Therefore, while it may be a contributing pathogenicity factor, it is possible to speculate that they are unlikely to account for the observed pathology.

It is critical to note that *ccaA* mutant exhibits a “run” bias in its chemotaxis phenotype. It is possible to hypothesise that the up-regulation of some genes in the *ccaA* isogenic mutant may occur to compensate for changes in chemotactic run-bias phenotype of the *ccaA* mutant. This lack of ability to tumble may indeed be the main cause of attenuation, as well as the reason for increase in invasiveness *in vitro*^31^ and bacterial presence in systemic organs.

*In vitro* cell culture assays using human cell cultures; Caco-2^31^, HepG2 and A549 cells, have shown that the *ccaA* mutant is more adherent and highly invasive compared to the wild-type. Although A549 is a human lung cell line, it contains α2,6-sialyl structures similar to those found in the chicken gastrointestinal tract^37,38^. The hyperinvasive phenotype of the *ccaA* mutant in A549 cells indicated that this might be similar to the events in the avian gastrointestinal tract.

In addition, the *ccaA* isogenic mutant was found to have significantly reduced ability to bind type II fucosylated structures, ubiquitous in all eukaryotic tissue types. A significant decrease in binding ability to structures including lacto-*N*-fucopentaose II, Lewis^y^, blood group H type II trisaccharide and monofucosyllacto-*N*-hexaose III was also observed. In contrast, *ccaA* isogenic mutant had significantly stronger binding to both 3′-sialyllactosamine and 6′-sialyllactosamine. The change in this binding ability, together with the “run” phenotype, may provide some insight into the presence of the mutant in the lung of chickens, as terminal αNeuAc2-3Gal structures are found predominantly in the lung of chickens^39^. This was evident by increased infection of A549 cells by the *ccaA* mutant, which could be due to increased expression of this glycan by the A549 cells.

These changes in glycan binding specificity of the *ccaA* mutant may contribute toward the pathology observed in model animals. The mutant bacteria may be able not only to move unidirectionally, but also be able to bind glycan structures differently in the avian gut, helping the bacteria to transverse the epithelium. In addition, previous studies have shown that binding to 3′- and 6′-sialyllactosamine structures by 11168-O wild-type cells, was significantly stronger at lower temperatures, 25 °C *in vitro*^40^. The higher core temperature of the chicken, 42 °C, the temperature at which the wild-type and mutant cells were tested using glycan arrays in this study, may account for the lower level of sialylated glycan binding for the wild-type compared to the *ccaA* mutant cells.

This study is the first report of gross pathological differences due to infection with an isogenic mutant, whereby mesenteric tissue abnormalities were observed within 72 hours p.i. In humans, a characteristic feature of Crohn’s disease is mesenteric adipose tissue hypertrophy, or fat wrapping (Reviewed in^41^). Mesenteric adipose tissue is an endocrine system and is able to regulated metabolic function and inflammation^41^. White adipose tissue is known to synthesise PPAR-γ and TNF-α, and release cytokines including adiponectin and IL-6^42,43^, hence may play a role in inflammatory response in Crohn’s disease. In chickens, IL-6 mRNA expression increases ten-fold at day 4 of colonisation^28^, and this may be more evident in chickens infected with the *ccaA* isogenic mutant, as in the mutant, an increase in mesenteric fat may also stimulate release of this cytokine. Although it appears that an inflammatory response is occurring in the chicken, it is unclear as the actual cause of the mesenteric adipose tissue hypertrophy. It is unusual, as in many diseases (including Crohn’s disease) as this manifests over many months, not days. Infectious colitis due to bacterial infection, including *Shigella*, *Salmonella*, *Yersinia*, *Campylobacter*, *Staphylococcus* and *Chlamydia*, have presented symptoms in humans including inflammatory changes in mesenteric fat^44^. Thus, this suggests that the increase in mesenteric fat may be a clinical sign of this *Campylobacter* infection.

Gross pathological changes were also observed in the murine model, when mice were infected with the *ccaA* mutant. The liver weight increased by at least 23% within 48 hours of infection in every mouse infected with the *ccaA* mutant. In addition, this persisted until at least day 6, even though there was no mutant bacterium present in the gastrointestinal tract at this time point. Similar to the findings of Vučković *et al*.^45^ hepatomegaly was observed in BALB/c mice, however there was no spleen enlargement, no yellowish nodes on the liver and no local tissue necrosis of the liver was observed in enlarged livers^45^. Unlike the study by Vučković^45^, where mice were infected with *C*. *jejuni via* intraperitoneal injection, in our study the mice were infected with *C*. *jejuni* by oral inoculation and the method of inoculation may influence the disease pathology observed in the mouse.

It is well established that one of the main roles of the liver is to remove bacteria from the blood stream and to resist infections by producing immune factors^46^. During inflammatory response to infection, the primary cellular reaction is the stimulation of monocytes and tissue macrophages^46^. Histological analysis revialed no statistical difference in cellular response in the livers of mice infected with the *ccaA* mutant compared to uninfected mice. Analyses of cytokine levels in mock-infected, 11168-O and *ccaA* mutant infected mice also showed no statistical difference in the pro-inflammatory cytokines at 24, 48 or 72 h time points. Since the mediators assessed did not change it is therefore unlikely that they contributed to the effect of gross pathological changes that were observed in the mouse.

Interestingly, the mice infected with the *ccaA* mutant showed enlarged mesenteric lymph nodes, which is a common feature of Crohn’s disease or ulcerative colitis^47^. However both groups of mice infected with *C*. *jejuni* had hyperplastic Peyer’s patches. The symptom of hyperplastic Peyer’s patches, where they project into the gut lumen as submucosal elevations, along with the enlargement of the liver, which is observed in the mice infected with the *ccaA* mutant, are typical symptoms of Typhoid or enteric fever^48,49^. *Campylobacter* is one of the non-salmonella organisms causes of infection which is clinically indistinguishable from classic enteric fever caused by *S*. *typhi*, *S*. *paratyphi* and *S*. *choleraesuis*^50,51^. There is little data on the pathological features of the associated enteritis, but similarities may exist between *Campylobacter* and *Salmonella* spp, as *C*. *jejuni* may access the submucosa via uptake by M cells, however it is unknown if *C*. *jejuni* can translocate across the cells in the colon^52,53^.

This study highlights the importance of an alteration of the phenotype due to irreversibly blocking or altering key colonisation factors, and the reason that they must be fully investigated. These factors are often potential targets for the development of new antimicrobial agents. Although there is a reduction in colonisation potential using both avian and mammalian colonisation models, one must ensure that there are no gross pathological changes occurring in the host due to this infection.

## Methods

### Bacterial strains and plasmids

The *C*. *jejuni* NCTC11168-O original strain was kindly donated by D.G Newell, VLA, London and its isogenic mutant strain 11168-O*ΔccaA::cat*^31^ were grown on Columbia agar supplemented with 5% defibrinated horse blood (HBA) with Skirrow antibiotic supplement (Oxoid) or Muellar Hinton agar (MHA) (Oxoid) under microaerophilic conditions (5% O_2_, 15% CO_2_, 80% N_2_; BOC gases) for 48 h at 37 °C or 42 °C where appropriate.

### Motility assays

Motility assays were performed as described by King *et al*.^54^. The motility phenotypes of *C*. *jejuni* 11168-O and 11168-O*ΔccaA::cat* were ascertained by calculating the spatial displacement of single cells. Singular cells were tracked via time lapse photography using ImageJ, over a one second time frame.

### Ethics Statement

Animal experiments were carried out in strict accordance with the Griffith University Animal Ethics Committee guidelines and assigned approval numbers MSC/04/08/AEC, BDD/01/07 and GLY/02/15/AEC. All procedures involving animals were reviewed and approved by National Health and Medical Research Council Australian code of practice for the care and use of animals for scientific purposes, 7th edition 2004.

### Avian and murine models

Unvaccinated Ross breed chickens (Bartters, Rochdale Qld) at one day after hatching were placed into groups of ten, pre-inoculation faecal samples were taken from the cloaca of the chickens and cultured. Chickens were housed in clean barrier cages at 32 °C and supplied sterilised food and water *ad libitum* for the entire experimental period. 129 × 1/SvJ background male mice (Animal Resource Centre, Western Australia) aged between 6–8 weeks, were housed in groups of 6–10 as specified, supplied *ad libitum* with food and water.

The animals were inoculated by orally challenging with 30 μL of Brucella broth containing approximately 1 × 10^8^ *C*. *jejuni* cells, as previously published^31^. Post-inoculation cloacal or faecal samples were taken daily and cultured. 24–96 h post-inoculation, as specified, the animals were euthanized by cervical dislocation, and the gastrointestinal tissues including small and large intestine as well as the systemic organs, lungs, liver and spleen, were removed aseptically. Each organ was weighed and apportioned. An appropriate portion was placed in a 5 mL tube with 2 mL of sterile Brucella broth, homogenised and the bacterial load enumerated by viable count. Tissue samples for histology were prepared using a portion of organ (systemic) or half of the longitudinally cut organ (digestive), fixed immediately in formal saline solution for at least 24 h. The samples were prepared for histology as described in Stahl *et al*.^55^.

### Histopathological scoring of organ tissue

The H & E stained sections were randomly coded for blind scoring, and the pathology of each organ was microscopically examined at 600 x magnification. The lungs, liver and spleen were examined for abnormalities including increase in leukocytes, tissue damage or changes in cellular arrangement. The digestive organs were examined in sections; small intestine, caecum, and proximal, mid and distal colon, for abnormal crypt architecture, crypt length, damage, goblet cell loss or hyperplasia, inflammatory cell infiltrate or the presence of neutrophils in the lamina propria. After scoring of each organ was performed, the samples were decoded and the results analysed.

### Direct *in vivo* Isolation of *C*. *jejuni* using M-280 Dyna-Beads

Immunomagnetic separation (IMS) of *C*. *jejuni* from mouse intestinal content or chicken caecal content was performed as previously described by King *et al*.^34^.

### Glycan Array analysis

IMS isolated cells were labelled with CFDA-SE and glycan binding profile was analysed as described in Day *et al*.^56^. Full list of glycan structures see Table S6.

### Adherence and Invasion Assays

The assays were performed according to^22^, with the modifications as described in^31^. The cell lines used in this study were A549 Human lung adenocarcinoma epithelial cell line (ATCC); Hep-G2 Human hepatocellular liver carcinoma cell line (ATCC).

### Cytometric bead array for quantification of inflammatory cytokines in mice

Mice were orally inoculated with Brucella broth, *C*. *jejuni* 11168-O or *C*. *jejuni* 11168-O*ΔccaA::cat* at 5 × 10^8^ CFU. After 24 hours, submandibular punctures were performed to remove approximately 100 µL of blood. Blood was allowed to coagulate for 15 mins, prior to centrifugation to separate serum. The BD CBA inflammatory mouse kit was used according to manufacturer’s instructions. After the beads were washed, data was acquired using a Beckman Coulter Cyan Flow cytometer, using the 488 nm laser with settings for FITC, APC and PE. Data was analysed using FlowJo software. Concentrations of cytokines were extrapolated from standard curves using Microsoft Excel.

### qPCR primers and primer design

*C*. *jejuni* primers were designed based on the published nucleotide sequence of *C*. *jejuni* 11168^57^. All primers used in this study are listed in Table S7, and source noted. Bacterial 23 s RNA primers or chicken 28 s RNA primers were used for internal controls. All oligonucleotide primers were synthesised by Invitrogen. RNA extraction, cDNA preparation and qPCR were performed as previously described^30^. A PCR standard curve was generated for each primer set by performing five ten-fold serial dilutions. Quantity values (copies) for gene expression was generated by comparison of the fluorescence generated by each sample with a standard curve of known quantities for each PCR product (Table S8).

### Quantification of inflammatory cytokine expression in chickens

Total RNA was extracted from the chicken small intestines from chicks inoculated with Brucella broth (negative), *C*. *jejuni* 11168-O or *C*. *jejuni* 11168-O*ΔccaA::cat*. The extracted RNA was used as template for the reverse transcription reaction. qPCR was performed using gene specific sense and anti-sense primers for specific chicken cytokines. All qPCR reactions were carried out using the same thermal profile conditions, 94 °C for 5 minutes, then 45 cycles of 94 °C for 30 seconds, 48 °C for 30 seconds then 72 °C for 1 minute, 30 seconds with fluorescence measured during the 72 °C extension phase. Melt curves for each amplification product were measured 80 times over the incremental increases in temperature. Products were visualised by agarose gel electrophoresis.

### Statistical analysis

The mean of the groups for bacteria load in each organ (n ≥ 5) were individually compared to that of control groups at the same time point. Significance was determined by un-paired t-tests with an alpha of 0.05. Analysis of variance (ANOVA) was performed to determine significance in relative gene expression in conjunction with an un-paired t-test. Histopathological scores were analysed by chi-square, non-parametric tests.

## Electronic supplementary material

Supplementary information

Supplementary data
