# Supplementary material for: A peculiar case of Campylobacter jejuni attenuated aspartate chemosensory mutant, able to cause pathology and inflammation in avian and murine model animals
Source: Sci Rep. 2018 Aug 22;8:12594. doi: 10.1038/s41598-018-30604-5 (PMC6105663; doi:10.1038/s41598-018-30604-5)
Supplement: Supplementary file 1 — Supplementary information [file 41598_2018_30604_MOESM1_ESM.docx]

**Supporting information captions.**

**S1 Table. Bacterial presence in systemic and digestive organs of mice infected with *C. jejuni* strains.** N = 9 or 10 mice. 0/10: no viable bacteria obtained from direct plating of organ homogenate.

**S2 Table. Pathology scoring of H&E stained histological sections from the intestines of mice infected with *C. jejuni* strains 11168-O*ΔccaA::cat* and 11168-O, or non-infected controls (Negative).** N = 2-10 mice

**S3 Table. Motility assessment.** Total linear displacement of bacterial cells was determined by single cell tracking via fluorescent microscopy. Total displacement (D_t_) = √(D_x_)^2^ + (D_y_)^2^, where D_x_ is final position_x-axis_ minus start position_x-axis_, and D_y_ is final position_y-axis_ minus start position_y-axis_. Data represents the average and standard deviation of 12 cell traces per strain. The average diameter (Ø) was measured from a swarm plate assay, where the diameter of bacteria after 24 hours. Data represents the average data and standard deviation from three replicates.

**S4 Table. Bacterial counts within organs of chickens infected with *C. jejuni* strains 11168-O*ΔccaA::cat* and 11168-O, or non-infected controls (Negative).** 1/10: one chicken out of ten positive for *Campylobacter* from direct plating of organ homogenate. N= 8-10 chicks

**S5 Table. Pathology scoring of H&E stained histological sections from the intestines of chickens infected with *C. jejuni* strains 11168-O*ΔccaA::cat* and 11168-O, or non-infected controls (Negative).** Inflammatory cell infiltrate: increased leukocytes in lamina propria. Villus epithelium shedding: noticeable damage to tip of villi. 1/5: one positive result from 5 slides analysed.

**S6 Table. Glycan array composition.**

**S7 Table. Primers used in this study.**

**S8 Table. Standard curve equations**.

**Figure S1. Expression of virulence genes *porA, peb1A* and *cdtABC.*** Expression levels of known virulence genes *porA, peb1A* and *cdtA, B* and *C,* from bacteria grown *in vitro* at 37^o^C, 42^o^C, and isolated by IMS *in vivo* from chicken and murine hosts. *C. jejuni* 11168-O WT 37^o^C;  *C. jejuni* 11168-O*ΔccaA::cat* 37^o^C; *C. jejuni* 11168-O WT 42^o^C;  *C. jejuni* 11168-O*ΔccaA::cat* 42^o^C; *C. jejuni* 11168-O WT chicken;  *C. jejuni* 11168-O*ΔccaA::cat* chicken;  *C. jejuni* 11168-O WT mouse;  *C. jejuni* 11168-O*ΔccaA::cat* mouse.
