# Supplementary material for: A peculiar case of Campylobacter jejuni attenuated aspartate chemosensory mutant, able to cause pathology and inflammation in avian and murine model animals
Source: Sci Rep. 2018 Aug 22;8:12594. doi: 10.1038/s41598-018-30604-5 (PMC6105663; doi:10.1038/s41598-018-30604-5)
Supplement: Supplementary file 2 — Supplementary data [file 41598_2018_30604_MOESM2_ESM.pdf]

A peculiar case of *Campylobacter jejuni* attenuated aspartate chemosensory mutant, able to cause pathology and inflammation in avian and murine model animals.

L.E. Hartley-Tassell, C.J. Day, E.A. Semchenko, G. Tram, L.I. Calderon-Gomez, Z. Klipic, A. M. Barry, A.K. Lam, M.A. McGuckin, V. Korolik.

Supplementary Table 1 Bacterial presence in systemic and digestive organs of mice infected with *C. jejuni* strains.

C. jejuni strain	Organ	24 hours	48 hours	72 hours	96 hours
11168*ΔccaA::cat*	Lungs	0/10	1/10	0/10	1/10
	Liver	1/10	2/10	1/10	1/10
	Spleen	0/10	0/10	0/10	0/10
	Small intestine	9/9	8/10	10/10	8/10
	Large intestine	10/10	10/10	9/10	10/10
11168-O	Lungs	0/10	0/10	0/10	0/10
	Liver	0/10	0/10	0/10	0/10
	Spleen	0/10	0/10	0/10	0/10
	Small intestine	3/10	4/10	7/10	8/10
	Large intestine	7/10	9/10	9/10	10/10
Uninfected	Lungs	0/10	0/10	0/10	0/10
	Liver	0/10	0/10	0/10	0/10
	Spleen	0/10	0/10	0/10	0/10
	Small intestine	0/10	0/10	0/10	0/10
	Large intestine	0/10	0/10	0/10	0/10

N = 9 or 10 mice. 0/10: no viable bacteria obtained from direct plating of organ homogenate

Supplementary Table 2 Pathology scoring of H&E stained histological sections from the intestines of mice infected with *C. jejuni* strains 11168-O*ΔccaA::cat* and 11168-O, or non-infected controls (Negative).

Pathology	24 hours			48 hours			72 hours			96 hours		
	**11168-O**	**11168-O*ΔccaA::cat***	**Negative**	**11168-O**	**11168-O*ΔccaA::cat***	**Negative**	**11168-O**	**11168-O*ΔccaA::cat***	**Negative**	**11168-O**	**11168-O*ΔccaA::cat***	**Negative**
**Small intestine**												
Goblet cell loss (>10%)	2/3	4/10	1/2	2/3	8/10	1/3	1/3	6/10	1/3	2/3	7/10	1/3
Lamina propria neutrophils	0/3	0/10	1/2	1/3	4/10	0/3	0/3	1/10	1/3	1/3	0/10	0/3
Inflammatory cell infiltration	2/3	9/10	1/2	2/3	8/10	0/3	2/3	6/10	1/3	2/3	5/10	0/3
**Large intestine**												
Goblet cell loss (>10%)	3/3	10/10	2/2	2/3	10/10	3/3	2/3	10/10	3/3	3/3	10/10	3/3
Goblet cell hypertrophy	2/3	5/10	2/2	2/3	5/10	0/3	2/3	5/10	3/3	2/3	7/10	2/3
Lamina propria neutrophils	3/3	10/10	2/2	3/3	10/10	3/3	3/3	7/10	2/3	2/3	10/10	2/3
Inflammatory cell infiltration	3/3	10/10	2/2	2/3	10/10	3/3	2/3	10/10	2/3	2/3	10/10	2/3

N = 2-10 mice.

Supplementary Table 3 Motility assessment

	11168-O wild-type	11168-O *ΔccaA::cat*	11168-O *ccaA+/-* complement
D_t	30.45 ± 20.39	94.01 ±41.06	31.15 ±29.37
Ø (mm)	3.25 ± 0.5	7.25 ± 1.89	3.13 ± 0.25

Total linear displacement of bacterial cells was determined by single cell tracking of fluorescently labelled (CFDA-SE) *C. jejuni*. Time-lapse image acquisition was performed at 2ms intervals using Nikon Ti-E inverted microscope at Griffith Imaging & Image Analysis Facility. Total displacement (D_t) = $\sqrt{(D_x)^2 + (D_y)^2}$, where D_x is final $\text{position}_{x\text{-axis}}$ minus start $\text{position}_{x\text{-axis}}$, and D_y is final $\text{position}_{y\text{-axis}}$ minus start $\text{position}_{y\text{-axis}}$. Data represents the average and standard deviation of 12 cell traces per strain. The average diameter (Ø) was measured from a swarm plate assay, where the diameter of bacteria after 24 hours. Data represents the average data and standard deviation from three replicates.

Supplementary Table 4 Bacterial counts within organs of chickens infected with *C. jejuni* strains 11168-O*ΔccaA::cat* and 11168-O, or non-infected controls (Negative).

	24 hours			48 hours			72 hours			96 hours		
Organ	11168-O*ΔccaA::cat*	11168-O	Negative	11168-O*ΔccaA::cat*	11168-O	Negative	11168-O*ΔccaA::cat*	11168-O	Negative	11168-O*ΔccaA::cat*	11168-O	Negative
Lungs	10/10	9/9	0/10	10/10	9/9	0/10	10/10	6/9	0/10	8/8	9/9	0/10
Liver	1/10	0/9	0/10	0/8	3/9	0/10	0/10	1/9	0/10	0/8	0/9	0/10
Spleen	1/10	0/9	0/10	0/8	3/9	0/10	0/10	0/9	0/10	0/8	0/9	0/10
Small intestine	9/10	9/9	0/10	8/8	9/9	0/10	10/10	9/9	0/10	8/8	9/9	0/10
Large intestine	9/10	9/9	0/10	8/8	9/9	0/10	10/10	9/9	0/10	8/8	9/9	0/10

1/10: one chicken out of ten positive for *Campylobacter* from direct plating of organ homogenate. N= 8-10 chicks

Supplementary Table 5 Pathology scoring of H&E stained histological sections from the intestines of chickens infected with *C. jejuni* strains 11168-O*ΔccaA::cat* and 11168-O, or non-infected controls (Negative).

Pathology	24 hours			48 hours			72 hours			96 hours		
	**11168-O**	**11168-O*ΔccaA::cat***	**Negative**	**11168-O**	**11168-O*ΔccaA::cat***	**Negative**	**11168-O**	**11168-O*ΔccaA::cat***	**Negative**	**11168-O**	**11168-O*ΔccaA::cat***	**Negative**
**Small intestine**												
Inflammatory cell infiltration	0/3	0/5	0/2	0/3	1/5	0/2	0/3	1/5	0/2	3/3	0/5	1/2
Villus epithelium shedding	1/3	4/5	2/2	2/3	5/5	2/2	1/3	4/5	2/2	3/3	5/5	1/2
Goblet cell loss (>10%)	0/3	0/5	0/2	0/3	0/5	0/2	0/3	0/5	0/2	0/3	0/5	0/2
**Large intestine**												
Inflammatory cell infiltration	0/3	0/5	1/2	0/3	1/5	0/2	0/3	0/5	0/2	0/3	0/5	1/2
Villus epithelium shedding	1/3	2/5	1/2	0/3	3/5	2/2	0/3	1/5	2/2	0/3	4/5	0/2
Goblet cell loss (>10%)	0/3	0/5	1/2	0/3	0/5	0/2	0/3	1/5	0/2	0/3	0/5	0/2

Inflammatory cell infiltrate: increased leukocytes in lamina propria. Villus epithelium shedding: noticeable damage to tip of villi. 1/5: one positive result from 5 slides analysed.

Supplementary Table 6 Glycan array composition

Code	Structure
	**Terminal Galactose**
1A	Lacto-*N*-Biose I
1B	*N*-Acetyllactosamine
1C	β-1-4-galactosyl-galactose
1D	β-1-6 Galactosyl-*N*-acetyl glucosamine
1E	β-1-3 Galactosyl-*N*-acetyl galactosamine
1F	β-1-3 Gal-*N*-Acetyl galactosaminyl- β1-4Gal-β1-4Glc
1G	Lacto-*N*-tetraose
1H	Lacto-*N*-neotetraose
1I	Lacto-*N*-neohexaose
1J	Lacto- *N* -hexaose
1K	Globotriose
1L	Tn Antigen GalNAca1-O-Ser
1M	Galactosyl-Tn Antigen
1N	α1-3 Galactobiose
1O	Linear B-2 Trisaccharide
1P	Linear B-6 Trisaccharide
2A	α1-3, β1-4, α1-3 Galactotetraose
2B	Gal β1-6Gal
2C	GalNAcβ1-3 Gal
2D	GalNAcβ1-4 Gal
2E	Galα1-4Gal β 1-4GlcNAc
2F	GalNAcα1-3Galβ1-4Glc

	**Terminal N`Acetyl glucosamine**
4A	*N*,*N*`-Diacetyl chitobiose
4B	*N*,*N*`,*N*``-Tiacetyl chitotriose

4C	*N*,*N*`,*N*``,*N*```-Tetraacetyl chitotetraose
4D	*N*,*N*`,*N*``,*N*```,*N*````-Hexaacetyl chitohexasose
4E	GlcNAcβ1-4MurNAc
	**Mannose containing structures**
5A	β1-2 *N* -Acetylglucosamine-mannose
5B	Bianntennary N-linked core pentasaccharide
5C	α1-2-Mannobiose
5D	α1-3-Mannobiose
5E	α1-4-Mannobiose
5F	α1-6-Mannobiose
5G	α1-3, α1-6-Mannobiose
5H	α1-3, α1-3, α1-6-Mannopentaose
	**Fucosylated structures**
7A	Lacto- *N* -fucopentaose I
7B	Lacto- *N* -fucopentaose II
7C	Lacto- *N* -fucopentaose III
7D	Lacto- *N* -difucohexaose I
7E	Lacto- *N* -difucohexaose II
7F	H-disaccaride
7G	2`-Fucosyllactose
7H	3`-Fucosyllactose
7I	Lewis^{x}
7J	Lewis^{a}
7K	Blood group A trisaccharide
7L	Lactodifucotetraose (LDFT)
7M	Blood group B trisaccharide
7N	Lewis^{y}
7O	Blood group H type II trisaccharide

7P	Lewis^{b} tetrasaccharide
8A	Sulpho Lewis^{a}
8B	Sulpho Lewis^{x}
8C	Monofucosyl-para-lacto- *N* -hexaose IV
8D	Monofucosyllacto- *N* -hexasose III
8E	Difucosyllacto- *N* -hexaose
8F	Tifucosyllacto- *N* -hexaose

Sialylated or Neu5Ac containing structures

10A	Sialyl Lewis^{a} (S Lea)
10B	Sialyl Lewis^{x} (S Lex)
10C	Sialyllactos- *N* -tetraose a
10D	Monosialyl, monofucosyllacto- *N* -neohexose
10K	3`-Sialyllactosamine
10L	6`-Sialyllactosamine
10M	LS-Tetrasaccharide a (LSTa)
10N	LS-Tetrasaccharide b (LSTb)
10O	LS-Tetrasaccharide c (LSTc)
10P	Disialyllacto- *N* -tetraose
11A	3`-Sialyllactose
11B	6`-Sialyllactose
11C	Colominic acid
11D	Biantennary 2,6-sialylated- *N* -glycan-Asn

Carageenan and Glycoaminoglycans (GAGs)

12A	Neocarratetraose-4^{1,3}-di-O-sulphate (Na^{+})
12B	Neocarratetraose-4^{1}-O-sulphate (Na^{+})
12C	Neocarrahexaose-2^{4},4^{1,3,5}-tetra-O-sulphate (Na^{+})
12D	Neocarrahexaose-4^{1,3,5}-tri-O-sulphate (Na^{+})
12E	Neocarraoctaose-4^{1,3,5,7}-tetra-O-sulphate (Na^{+})

12F	Neocarradecaose-$4^{1,3,5,7,9}$-penta-O-sulphate (Na^{+})
12G	ΔUA-2S-GlcNS-6S
12H	ΔUA-GlucNS-6S
12I	ΔUA-2S-GlucNS
12J	ΔUA-2S-GlcNAc-6S
12K	ΔUA-GlcNAc-6S
12L	ΔUA-2S-GlcNAc
12M	ΔUA-GlcNAc
12N	ΔUA-GalNAc-4S (Delta Di-4S)
12O	ΔUA-GalNAc-6S (Delta Di-6S)
12P	ΔUA-GalNAc-4S,6S (Delta Di-disE)
13A	ΔUA-2S-GalNAc-4S (Delta Di-disB)
13B	ΔUA-2S-GalNAc-6S (Delta Di-disD)
13C	ΔUA-2S-GalNAc-4S-6S (Delta di-tisS)
13D	ΔUA-2S-GalNAc-6S (Delta Di-UA2S)
13E	ΔUA-GlcNAc (Delta Di-HA)
13F	Hyaluronan fragment (4mer)
13G	Hyaluronan fragment (8mer)
13H	Hyaluronan fragment (10mer)
13I	Hyaluronan fragment (12mer)
13J	Heparin
13K	Chondroitin sulfate
13L	Dermatan sulfate
13M	Chondroitin 6-sulfate

Supplementary Table 7 Primers used in this study.

Primer name	5`-3` primer sequence.	Source
Tlp1p F	TTG TTA TCG TTT ACG CTG ATG	[1]
Tlp1p R	TGG AAG ATC TTT ATT ATA ATT TTT TAA GGG TTT AA	[1]
IL-8 chick F	GCC CTC CTC CTG GTT TCA G	[2]
IL-8 chick R	TGG CAC CGC CAG CTC ATT	[2]
CXCLi1 chick F	CCAGTGCATAGAGACTCATTCCAA	[3]
CXCLi1 chick R	TGC CCA TCT TTC AGA GTA GCT ATG AAC T	[3]
IL-6 chick F	GCT CGC CGG CTT CGA	[4]
IL-6 chick R	GGT AGG TCT GAA AGG CGA ACA G	[4]
IL-1β chick F	GCT CTA CAT GTC GTG TGT GAT GAG	[5]
IL-1β chick R	TGT CGA TGT CCC GCA TGA	[5]
TNF-α chick F	GAC TAG GCT CCC ACA CTA CC	[6]
TNF-α chick R	ACT GGG CGG TCA TAG AAC AG	[6]
INF_{γ} chick F	GTG AAG AAG GTG AAA GAT ATC ATG GA	[5]
INF_{γ} chick F	GCT TTG CGC TGG ATT CTC A	[5]
CdtA F	ATG CAA AAA ATT ATA GTT	[7]
CdtA R	TCA TCG TAC CTC TCC TTG	[7]
CdtB F	ATC TTT TAA CCT TGC TTT TGC A	[7]
CdtB R	TTG CGC TAG TTG GAA AAA CCA CT	[7]
CdtC F	GGA GAT TTG AAA GAT TTT AGG G	[7]
CdtC R	CAT CTT GAC AAG ATT TTG CTC C	[7]
PorA F	TGG ACG GAT AAC GCT ATT G	[7]
PorA R	TTG CCA TTA GCG TGT GTT TT	[7]

Peb1A F	AGC AAT GCT AAT GCA GCA GA	[7]
Peb1A R	AGT TGC AGC TTG AGC CAC TC	[7]
28S RNA F	GGC GAA GCC AGA GGA AAC T	[4]
28S RNA R	GAC GAC CGA TTT GCA CGT C	[4]
23S RNA F	TTA TCC AAT ACC AAC ATT AGT	[8]
23S RNA R	GAA GAT ACG GTG CTA TTT TG	[8]

1 Hartley-Tassell, L. E. *et al.* Identification and characterization of the aspartate chemosensory receptor of *Campylobacter jejuni*. *Mol Microbiol* **75**, 710-730, doi:MMI7010 [pii] 10.1111/j.1365-2958.2009.07010.x (2010).

2 Shini, S. & Kaiser, P. Effects of stress, mimicked by administration of corticosterone in drinking water, on the expression of chicken cytokine and chemokine genes in lymphocytes. *Stress* **12**, 388-399, doi:10.1080/10253890802526894 (2009).

3 Withanage, G. S. K. *et al.* Rapid Expression of Chemokines and Proinflammatory Cytokines in Newly Hatched Chickens Infected with Salmonella enterica Serovar Typhimurium. *Infection and Immunity* **72**, 2152-2159, doi:10.1128/IAI.72.4.2152-2159.2004 (2004).

4 Smith, C. K. *et al.* Campylobacter jejuni-induced cytokine responses in avian cells. *Infect Immun* **73**, 2094-2100, doi:10.1128/IAI.73.4.2094-2100.2005 (2005).

5 Kaiser, P. *et al.* Differential cytokine expression in avian cells in response to invasion by Salmonella typhimurium, Salmonella enteritidis and Salmonella gallinarum. *Microbiology* **146 Pt 12**, 3217-3226, doi:10.1099/00221287-146-12-3217 (2000).

6 Tabatabaei, S., Badalzadeh, R., Mohammadnezhad, G. & Balaei, R. Effects of Cinnamon extract on biochemical enzymes, TNF–α and NF-κB gene expression levels in liver of broiler chickens inoculated with *Escherichia coli*. *Pesq. Vet. Bras* **35**, 781-787 (2015).

7 King, R. M. *et al.* Carbohydrate binding and gene expression by in vitro and in vivo propagated Campylobacter jejuni after immunomagnetic separation. *Journal of basic microbiology* **53**, 240-250, doi:10.1002/jobm.201100466 (2013).

8 Eyers, M., Chapelle, S., Van Camp, G., Goossens, H. & De Wachter, R. Discrimination among thermophilic *Campylobacter* species by polymerase chain reaction amplification of 23S rRNA gene fragments. *Journal of Clinical Microbiology* **32**, 1623 (1994).

Supplementary Table 8 Standard curve equations

Gene	Standard curve equation	Primer efficiency
Tlp1	y = 3.764 + 42.062	84.3%
IL-8	y = 1.925 + 0.206	62.1%
CXCLi1	y = 3.180 + 1.017	88.1%
IL-1β	y = 3.339 + 26.991	82.8%
IL-6	y = 4.664 + 27.998	77.9%
TNFα	y = 2.864 + 16.733	93.2%
IFN_{γ}	y = 9.131 + 14.809	93.0%
PorA	y = 4.805 + 49.732	61.5%
Peb1A	y = 5.712 + 51.516	49.6%
CdtA	y = 4.408 + 46.408	69.0%
CdtB	y = 4.738 + 55.271	62.6%
CdtC	y = 2.281 + 36.538	63.6%
23S RNA	y = 3.828 + 43.454	82.1%
28S RNA	y = 3.428 + 2.316	93.%

Figure S1. Expression of virulence genes *porA, peb1A* and *cdtABC.*

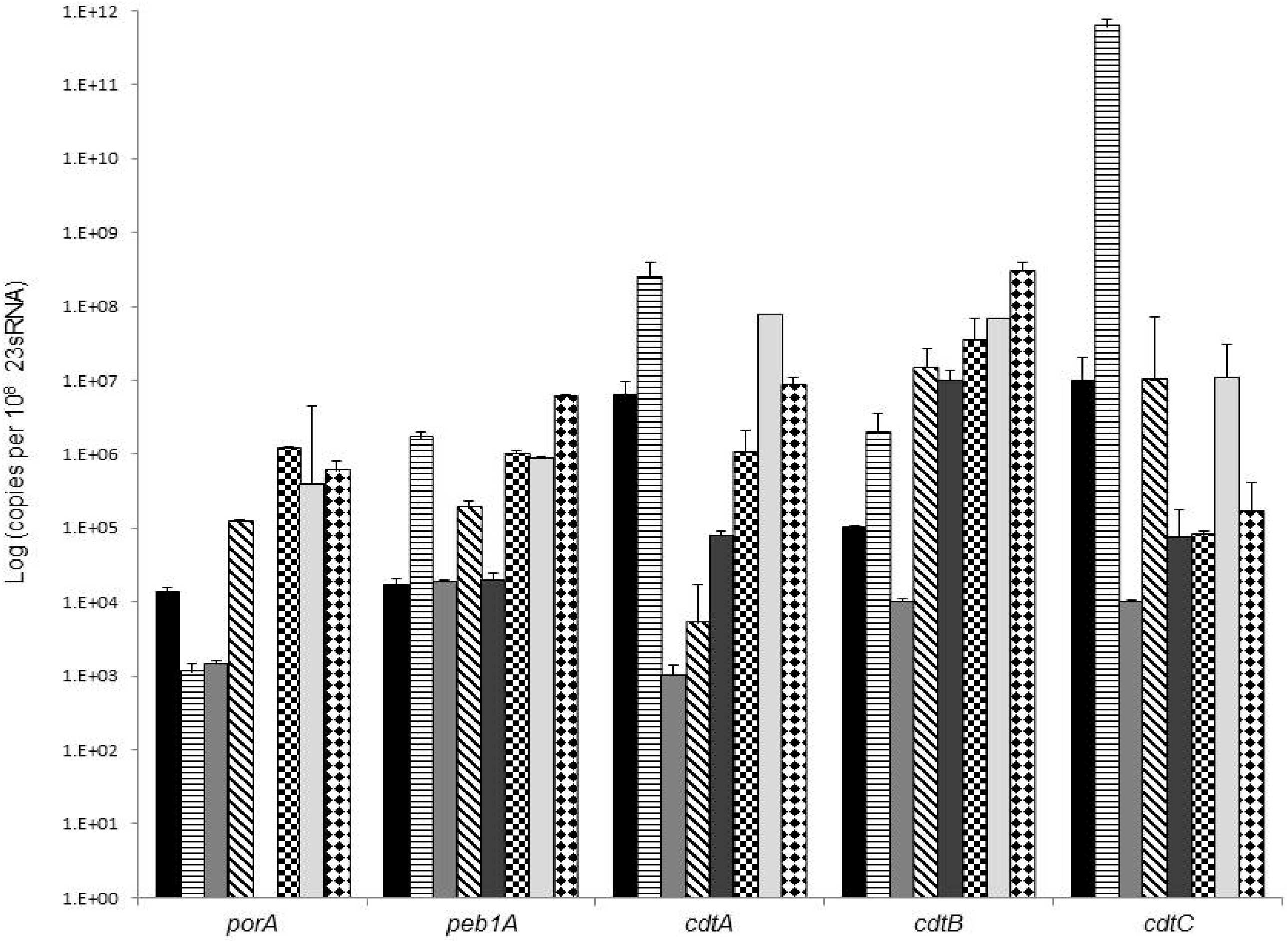

Expression levels of known virulence genes *porA*, *peb1A* and *cdtA*, *B* and *C*, from bacteria grown *in vitro* at 37°C, 42°C, and isolated by IMS *in vivo* from chicken and murine hosts. *C. jejuni* 11168-O WT 37°C; *C. jejuni* 11168-O*ΔccaA::cat* 37°C; *C. jejuni* 11168-O WT 42°C; *C. jejuni* 11168-O*ΔccaA::cat* 42°C; *C. jejuni* 11168-O WT chicken; *C. jejuni* 11168-O*ΔccaA::cat* chicken; *C. jejuni* 11168-O WT mouse; *C. jejuni* 11168-O*ΔccaA::cat* mouse.
